# Clinical and Technical Considerations for Low-Level Laser Therapy in Temporomandibular Disorders: A Narrative Review

**DOI:** 10.7759/cureus.117795

**Published:** 2026-10-12

**Authors:** Nezha Ben El Hammi, Fatima Zahra Amessegher, Kaotar Eddaouri, El Mehdi Jouhadi

**Affiliations:** 1 Department of Fixed Prosthodontics, Faculty of Dentistry Casablanca, Hassan II University, Casablanca, MAR

**Keywords:** dental lasers, low-level laser therapy, orofacial pain disorders, photobiomodulation, tmds (temporomandibular joint disorders)

## Abstract

Temporomandibular disorders (TMD) are common, often heterogeneous musculoskeletal conditions involving the temporomandibular joint, the masticatory muscles, and associated structures, and they are a leading cause of nondental orofacial pain. Low-level laser therapy (LLLT), also known as photobiomodulation (PBM), has attracted growing interest as a conservative, noninvasive option because of its analgesic and anti-inflammatory effects. This narrative review focuses on the practical technical parameters of LLLT, including wavelength, fluence, output power, emission mode, application sites, session number and frequency, and irradiation time per point, and on its clinical effects on the principal symptoms of TMD. A narrative search of PubMed, Scopus, Google Scholar, and ScienceDirect was conducted, prioritizing systematic reviews, meta-analyses, randomized controlled trials, and clinically relevant studies reporting technical parameters and outcomes. Because the marked heterogeneity of diagnostic criteria is a principal source of variability in this literature, the review also emphasizes the Diagnostic Criteria for Temporomandibular Disorders (DC/TMD) and the distinction between myogenic, arthrogenic, and mixed subtypes, whose optimal parameters are likely to differ. Current evidence suggests that LLLT may reduce pain intensity, improve maximum mouth opening, and decrease muscular tender points and joint sounds in selected patients; however, the magnitude of these effects varies considerably across studies, reflecting substantial heterogeneity in diagnostic criteria, dosimetric protocols, and outcome measures, and these findings should not be interpreted as a uniformly predictable clinical response. Near-infrared wavelengths of approximately 810 to 1100 nm are supported, although the optimal subrange differs between reviews (810 to 940 nm vs. 910 to 1100 nm); reported protocols commonly use energy densities of 3 to 12 J/cm² and three to 12 sessions applied one to three times per week, with highly variable irradiation times per point, mainly targeting the temporomandibular joint region and the masseter and temporalis muscles. In the limited number of active-comparator trials, generally short-term, LLLT appeared comparable or superior to occlusal splints, nonsteroidal anti-inflammatory drugs (NSAIDs), and transcutaneous electrical nerve stimulation (TENS), and it was well-tolerated, with only rare and transient adverse effects. However, considerable heterogeneity persists regarding diagnostic criteria, dosimetry reporting, device characteristics, treatment duration, and follow-up, and this variability, rather than a consistent dose-effect relationship, is a central finding of the present review. LLLT may therefore be a useful adjunctive treatment for TMD within multidisciplinary conservative management, but standardized, fully reported dosimetric protocols and high-quality randomized trials are needed to determine optimal parameters by TMD subtype, chronicity, and patient characteristics.

## Introduction and background

Temporomandibular disorders (TMD) refer to a group of musculoskeletal conditions characterized by pain and/or dysfunction of the masticatory muscles (the muscles involved in chewing and jaw movement), the temporomandibular joint (TMJ), and associated structures [1]. They represent the most common cause of nondental orofacial pain and carry a significant risk of progressing to chronicity. Their global prevalence is estimated at approximately 34%, with notable regional variations: reaching 47% in South America, 33% in Asia, and 29% in Europe. Adults aged 18 to 60 are the most affected (41%), and a clear female predominance is observed. These figures make TMD a worldwide public health issue. Their pathophysiology fits within a biopsychosocial model, integrating closely intertwined biological, psychological, and social factors. Early diagnosis and appropriate management are essential to improve functional outcomes and patients’ quality of life [2].

The management of TMD is structured around four principal therapeutic axes: conservative approaches (physiotherapy, pharmacotherapy, and injectable treatments), orthopedic devices (notably occlusal splints), corrective structural interventions (prosthetic rehabilitation, orthodontics, and maxillofacial surgery), and preventive or interceptive strategies [1,3].

Among conservative treatments for TMDs, low-level laser therapy (LLLT), also referred to as photobiomodulation (PBM), has emerged as a promising non-invasive therapeutic modality. This technique involves the application of light at a specific wavelength, generally in the red or near-infrared spectrum, to painful or injured tissues [4]. Unlike surgical lasers, which act primarily through thermal and ablative effects, LLLT, also known as PBM, relies on a nonthermal, photochemical mechanism. Photons are absorbed by endogenous chromophores, most notably cytochrome c oxidase, located in the mitochondrial membrane. This process can be loosely compared to cellular photosynthesis, in that it uses light energy to enhance an existing cellular energy production pathway rather than to generate new chemical energy. This absorption triggers a cascade of biological effects at the cellular and tissue levels: increased ATP production through accelerated electron transfer in the respiratory chain, modulation of reactive oxygen species (ROS) and nitric oxide (NO), increased intracellular calcium, and stimulation of cell proliferation and tissue repair. LLLT also promotes vasodilation and microcirculation, reduces inflammation and edema, and contributes to analgesia. These effects occur without a significant increase in tissue temperature, which distinguishes LLLT from thermal lasers [5,6]. This mechanism-based, nonpharmacological action distinguishes LLLT from more traditional conservative treatments for TMD, such as nonsteroidal anti-inflammatory drugs (NSAIDs), which act systemically and carry gastrointestinal and renal risks with prolonged use, or occlusal splints, which act primarily through mechanical rather than biological means, positioning LLLT as a potentially complementary, rather than substitutive, option within the conservative treatment armamentarium.

It should also be emphasized that, although PBM targets local nociceptive and inflammatory processes through peripheral mechanisms such as increased ATP production, improved microcirculation, reduced edema, and decreased prostaglandin E2 and cyclooxygenase-2 levels, it should be regarded as one component of a broader, multimodal management strategy. Multimodal approaches, including physical therapy, pharmacological treatment, occlusal splints, and other conservative interventions, appear to be more effective in patients with TMD, particularly when central sensitization and psychological factors contribute to chronicity within the biopsychosocial model of TMD [6]. 

This literature review examines the efficacy of LLLT in the management of TMD, with a specific focus on two aspects: (1) the technical parameters of application and treatment protocols, and (2) the clinical effects on the principal symptoms of TMD. Unlike previous reviews that have mainly focused on overall efficacy, this narrative review emphasizes the practical technical parameters of LLLT, including wavelength, fluence (the light energy delivered per unit area, expressed in J/cm²), application sites, session frequency, and treatment duration, to provide a clinically oriented synthesis for dental and orofacial pain practice. Despite growing clinical interest in LLLT for TMD, published protocols vary widely in wavelength, dose, and session number, and this heterogeneity in reporting makes it difficult for clinicians to translate existing evidence into a reproducible treatment protocol. Addressing this practical, protocol-level gap, rather than re-establishing overall efficacy, which has already been examined by prior reviews, is the specific aim of the present work.

Beyond TMD management, LLLT has broader applications in dentistry, including accelerating wound healing across oral soft tissues and promoting bone repair and remodeling [7], reflecting its recognized anti-inflammatory, analgesic, and biostimulatory effects across dental specialties [8,9]. Nevertheless, standardization of dosimetric parameters remains essential to optimize clinical outcomes across these applications [10]. 

A narrative, nonsystematic literature search was conducted using PubMed, Scopus, Google Scholar, and ScienceDirect to identify relevant articles on LLLT, PBM, and TMD. The search covered records from database inception to June 2026, and the included studies ranged in publication date from 2006 to 2026. The following keywords were used alone and in combination, without a single fixed Boolean string applied uniformly across all databases: "low level laser therapy," "photobiomodulation," "temporomandibular disorders," "temporomandibular joint," "orofacial pain," "laser therapy," and "pain management"; an example of the terms combined for PubMed was ("low level laser therapy" OR "photobiomodulation") AND ("temporomandibular disorders" OR "temporomandibular joint"). Only articles published in English were retained. Studies were included if they addressed: population, patients diagnosed with TMD (any subtype); intervention, LLLT/PBM; comparator, placebo, sham treatment, conventional therapies (occlusal splints, NSAIDs, transcutaneous electrical nerve stimulation (TENS)), or no comparator for studies focused on dosimetric/technical parameters; and outcome, pain intensity (VAS), mandibular function/mouth opening, tender points, or joint sounds. Case reports, non-peer-reviewed sources, and articles not published in English were excluded. Article selection and synthesis were performed narratively by the authors based on relevance and methodological quality, without a formal Preferred Reporting Items for Systematic Reviews and Meta-Analyses (PRISMA)-based screening flow diagram, risk of bias assessment, or meta-analytic pooling, consistent with the Scale for the Assessment of Narrative Review Articles (SANRA) criteria for narrative reviews [11]. Data extraction was performed by one author (N.B.), with dosimetric parameters and clinical outcomes cross-checked by the remaining co-authors; discrepancies were resolved by discussion among the authors. We emphasize that this review follows a selective, narrative methodology rather than a systematic one, and its findings should be interpreted as a qualitative, expert-guided synthesis of the literature rather than an exhaustive or quantitative evidence synthesis.

## Review

Diagnostic considerations

Before any consideration of LLLT, an explicit diagnostic framework is essential, as the marked heterogeneity of diagnostic criteria across studies constitutes a principal source of the variability observed in this literature. Contemporary practice relies on the Diagnostic Criteria for Temporomandibular Disorders (DC/TMD), which revised the earlier Research Diagnostic Criteria (RDC/TMD) and distinguishes twelve structural subcategories. These fall into two broad groups: muscular (myogenic) disorders, chiefly myalgia and its variants including myofascial pain and headache attributed to TMD, and articular (arthrogenic) disorders, ranging from arthralgia to disc-condyle displacements with or without reduction, and degenerative joint disease. In practice, the two frequently coexist as mixed musculo-articular presentations. This distinction is not merely nosological: superficial masticatory-muscle pain and deep condylar or disc pathology are unlikely to require the same penetration depth, wavelength, or application sites, so the optimal dosimetric parameters probably differ according to subtype. Reporting the diagnostic criteria used and the distribution of TMD subtypes is therefore a prerequisite for interpreting and comparing the outcomes of LLLT [1].

Mechanism of action and biological impact of LLLT

LLLT acts primarily through photon absorption by mitochondrial cytochrome c oxidase, leading to increased ATP production, a transient rise in reactive oxygen species, and the release of NO. These events trigger multiple intracellular signaling pathways that modulate cell proliferation, differentiation, and survival, with effects highly dependent on wavelength, fluence, and irradiation frequency following a biphasic dose-response pattern [12]. LLLT exerts a pronounced anti-inflammatory effect by reducing pro-inflammatory cytokines such as TNF-α, IL-1β, and IL-6, decreasing oxidative stress markers, and promoting the shift of macrophages from the pro-inflammatory M1 phenotype toward the reparative M2 phenotype [13,14].

At the analgesic level, several complementary mechanisms have been proposed to explain the pain-relieving action of LLLT. PBM reduces pro-inflammatory mediators such as prostaglandin E2 and cyclooxygenase-2 [6,14], inhibits nociceptive signals arising from peripheral nerves, and raises the pain threshold through effects on the cellular membrane potential [7,14]; it also improves local microcirculation and lymphatic drainage, which accelerates the resorption of edema [6,7]. In parallel, the stimulation of mitochondrial activity enhances tissue repair by promoting fibroblast proliferation, collagen synthesis, and angiogenesis [13]. Crucially, these effects follow the biphasic dose-response principle (the Arndt-Schulz law): insufficient energy produces no measurable photobiomodulatory effect, whereas excessive energy may become inhibitory, so that an optimal therapeutic window exists [12,14]. This dose dependence explains why apparently similar protocols can yield divergent clinical results and underscores the importance of reporting complete dosimetric parameters.

Technical parameters of LLLT in the treatment of TMDs

The technical parameters of LLLT in the treatment of TMD have been investigated in numerous clinical studies, although considerable heterogeneity remains between protocols.

Dosimetry and Laser Physics

Wavelength: Near-infrared wavelengths are the most frequently used for TMD pain management. Ren et al., in a network meta-analysis of 27 randomized controlled trials (RCTs) involving 969 patients, found that the 910-1100 nm band produced the greatest pain reduction compared with placebo immediately after treatment (mean difference: 4.68; 95% CI: 3.08-6.28), whereas at one-month follow-up, only the 780-904 nm band remained significantly superior to placebo (mean difference 1.59, 95% CI 0.07-3.10); TENS also reduced pain immediately after treatment, without a statistically significant difference compared with the infrared laser bands [15]. In addition, Carvalho et al. highlighted that infrared lasers in the 780 to 904 nm range (up to 980 nm) are the most suitable due to their superior tissue penetration compared to red lasers, and that efficacy is enhanced by high-energy density protocols and a high number of sessions [10].

Tissue optics help explain the preference for the near-infrared range: penetration depth is greatest in the lower portion of this range and decreases at longer wavelengths again as water absorption becomes more significant [14]. Clinically, however, the optimal subrange differs between reviews. Díaz et al. identified wavelengths between 810 nm and 940 nm as optimal for TMD pain management [16], whereas the network meta-analysis by Ren et al. ranked the 910-1100 nm band first, with the greatest pain reduction immediately after treatment, although, when this band was analyzed separately, this advantage was no longer statistically significant at one-month follow-up [15]. This discrepancy suggests that clinical efficacy cannot be explained by penetration depth alone, particularly as some protocols extend toward 980 nm despite the reduction in penetration beyond approximately 810 nm [10]. The emission mode and output characteristics of the devices studied also differ, so that the optimal wavelength reported in each source remains inseparable from its specific protocol and clinical target. Taken together, the evidence consistently supports the near-infrared range for TMD pain, although the optimal subrange varies between 810 and 1100 nm depending on the review and protocol considered.

Energy density (fluence): According to a systematic review of 44 RCTs involving 1,816 participants, energy densities between 3 and 12 J/cm² provided the most consistent results, whereas lower doses (<2 J/cm²) had negligible therapeutic effects [16]. Reported output power ranged from 0.03 W to 1 W, with the highest powers (0.5-1 W) yielding the best results in arthrogenic TMD [16]. The existence of an effective range, rather than a simple dose-dependent increase, reflects the biphasic dose response (Arndt-Schulz law) described above: fluences below the therapeutic window produce little or no photobiomodulatory effect, whereas fluences above it may become inhibitory, so that both underdosing and overdosing reduce clinical efficacy [12].

Irradiance and spot size: Irradiance (or power density), expressed in W/cm², corresponds to the average power output divided by the spot size of the laser probe and, together with exposure time, determines the delivered fluence. It was rarely reported in the RCTs included in the systematic review by Díaz et al., and the available values varied widely, from below 0.01 W/cm² to 0.4 W/cm² [16]. Spot size (beam area) is equally critical: because delivered energy equals fluence multiplied by the irradiated area, the same fluence corresponds to markedly different delivered energies depending on the spot size, which should therefore always be explicitly stated to allow accurate comparison and replication of protocols. To ensure reproducibility, these and the remaining dosimetric parameters are summarized in Table 1, following the reporting recommendations of the World Association for Photobiomodulation Therapy (WALT, formerly World Association of Laser Therapy) [17].

**Table 1:** Reported ranges of clinical parameters for low-level laser therapy in TMD TMJ: temporomandibular joint; TMD: temporomandibular disorder; RCT: randomized controlled trial These parameters are not definitive, standardized recommendations, as considerable heterogeneity remains across published protocols in wavelength, fluence, power output, number of sessions, and anatomical application sites

Parameter	Reported range	Clinical comment
Wavelength	810-1100 nm	Near-infrared wavelengths are preferred for pain reduction in TMD; the optimal subrange varies between reviews (810-940 nm vs. 910-1100 nm) [15,16]
Energy density (fluence)	3-12 J/cm²	Provided the most consistent results; doses below 2 J/cm² had negligible effects [16]. The energy dose should also be reported in joules [17]
Output power	0.03-1 W	The highest powers (0.5-1 W) yielded the best results in arthrogenic TMD [16]. The average output power should be reported [17]
Emission mode	Continuous wave or pulsed	Rarely reported; for pulsed emission, frequency (Hz), pulse duration, and duty cycle should also be stated
Spot size (beam area)	Device-dependent; rarely reported	Must be stated, since the same fluence corresponds to different delivered energies depending on spot area [17]
Irradiance (power density)	Rarely reported; <0.01-0.4 W/cm² in available RCTs	Equals average power divided by spot area; together with exposure time, determines the delivered fluence [16]
Energy per point	Rarely reported	Equals average power multiplied by irradiation time per point.
Total energy	Rarely reported	Equals energy per point multiplied by the number of irradiated points; the accumulated energy over all sessions should be reported [17]
Number of sessions	3-12 sessions	Multiple sessions are generally required to obtain sustained clinical benefits [16,18]
Frequency	1-3 sessions/week (up to 5)	Repeated applications over several weeks may provide longer-lasting outcomes than isolated sessions [16,18]
Treatment duration	Commonly 2-4 weeks (up to 6 months)	Protocols exceeding four weeks are associated with more sustained improvements [16,18]
Time per point	10-300 s (highly variable)	Depends on output power, target energy, and number of irradiated points [16,18]
Application sites	TMJ region, masseter, temporalis; in some protocols, cervical muscles and intra-oral pterygoid points	Often applied at trigger or tender points according to symptoms [18,19]
Mode of application	Contact or noncontact	Rarely reported; both direct skin contact and non-contact techniques have been described [18,19]
Main expected effects	Pain reduction, improved mouth opening, and fewer tender points	Clinical improvement may vary according to TMD subtype, chronicity, treatment protocol, and patient-related factors [16,19]

Clinical Protocol Execution

Application sites, session frequency, and treatment duration: Regarding application sites, the most frequently targeted sites are the temporomandibular joint region and the masticatory muscles, particularly the masseter and temporalis, often at trigger or tender points [18]. Some protocols extend the application around the joint (in front of, behind, and above it) and into the external acoustic meatus, to tender points in the sternocleidomastoid, trapezius, and posterior neck muscles, and intra-orally to the masseter, the temporalis attachment on the anterior border of the ramus, and the lateral and medial pterygoid muscles [19]. The number and frequency of sessions vary considerably between protocols. In the systematic review by Buduru et al., the number of sessions ranged from three to 12, applied once to three times per week, mostly over two to four weeks [18]. In the larger review by Díaz et al., frequencies ranged from two to five sessions per week and treatment durations from three weeks to six months, with protocols exceeding four weeks providing more sustained improvements [16]. Application time per point varies widely, ranging from 10 to 300 seconds in the systematic review by Buduru et al. [18] and up to 460 seconds among the RCTs included by Díaz et al., without a clearly predominant duration [16]. The mode of application is rarely specified; both direct skin contact [19] and noncontact techniques [18] have been described. Short treatment protocols may provide rapid pain relief, but these effects are often transient, whereas protocols exceeding four weeks are associated with more sustained improvements, suggesting a cumulative effect of PBM [16]. In summary, current evidence does not yet support a single standardized LLLT protocol for TMD [18], although protocols exceeding four weeks appear to provide more sustained benefits [16].

Clinical effects on principal symptoms of TMDs

The clinical effects of LLLT on the principal symptoms of TMD have been extensively documented across RCTs and systematic reviews. Regarding pain reduction, a systematic review of 44 RCTs comprising 1,816 participants confirmed that LLLT significantly decreases pain intensity, with a 60-70% reduction in VAS scores, demonstrating superior or comparable outcomes to conventional treatments such as occlusal splints, NSAIDs, and TENS therapy [16]. These analgesic effects have been attributed to the release of endogenous opioids, an increased pain threshold through effects on the cellular membrane potential, vasodilation and improved local microcirculation, reduction of edema through increased lymphatic flow, decreased prostaglandin E2 and cyclooxygenase-2 levels, and enhanced ATP production [6,7]. Concerning mandibular function and maximum mouth opening, the same systematic review reported a 10-20% increase in vertical aperture, with more sustained functional improvements observed in treatment protocols exceeding four weeks [16]. An updated systematic review of 40 studies including 1,927 patients further confirmed that seven out of fifteen studies reporting maximum mouth opening showed a significantly greater improvement in PBMT groups compared to placebo groups [20]. With respect to muscular tender points and joint sounds, a clinical study evaluating LLLT in 20 TMD patients using a gallium arsenide diode laser (904 nm, 4 J/cm², 6 sessions over 2 weeks) reported statistically significant reductions in the number of tender points and joint sounds, alongside improvement in the active range of jaw motion, with results maintained at the six-month follow-up [19]. A double-blind placebo-controlled trial further corroborated these findings, reporting significant improvements in pain reduction, maximum mouth opening, mandibular deviation, and articular clicking in the LLLT group compared to placebo, supporting LLLT as an effective nonsurgical adjunct in the management of TMDs [21].

These clinical findings should nonetheless be interpreted with methodological caution. Because LLLT is nonthermal [6] and near-infrared light is largely invisible, effective blinding is theoretically achievable; however, its quality varies across trials. Díaz et al. reported that some RCTs displayed inadequate randomization, lack of blinding, or high dropout rates [16], and Buduru et al. found a high risk of performance bias in two of the five RCTs assessed and a high risk of bias related to allocation concealment in most of them [18]. Moreover, because the analgesic outcomes are largely self-reported on the VAS and the active device is difficult to distinguish from a sham, part of the observed improvement may reflect a placebo or expectation effect [15]. Consistent reporting of the sham modality and of allocation concealment is therefore essential before the effect sizes reported in this literature can be taken at face value. Because allocation concealment was frequently unclear or at high risk of bias [15,18], and the certainty of the evidence for most comparisons was rated low to moderate according to the Grading of Recommendations Assessment, Development and Evaluation (GRADE) [15], the overall certainty of the evidence for LLLT's analgesic effect should be considered moderate at best, and this should temper the strength of any clinical recommendation drawn from this review.

Table 2 summarizes the principal systematic reviews, meta-analyses, and clinical trials on which this review draws. Across these studies, LLLT generally reduced pain relative to placebo, whereas effects on mandibular function were less consistent, with only seven of 15 studies reporting a significantly greater improvement in maximum mouth opening in one systematic review [20] and no between-group difference in mouth opening in a recent blinded trial [22]; the magnitude of effect also varied according to wavelength, delivered dose, and TMD subtype. Recent primary randomized trials using DC/TMD diagnostic criteria continue to inform this evidence base. One trial directly compared separately diagnosed myofascial (myogenic) and disk-displacement-with-reduction (arthrogenic) cohorts and found statistically significant pain reduction at one and six months in both groups; however, comparable improvements were also observed in a soft-diet-only control group, with no clear superiority of LLLT over the control condition, suggesting that part of the improvement may reflect nonspecific factors such as placebo response, patient expectations, or the natural course of the disorder [23]. Another recent blinded, placebo-controlled trial using DC/TMD Axis I and II diagnostic criteria confirmed a significant analgesic effect of LLLT relative to placebo [22]. Benefits have most consistently been reported in myogenic presentations and with near-infrared wavelengths (810 to 940 nm) applied at adequate cumulative doses [16], although emerging subtype-stratified data suggest that arthrogenic presentations may respond comparably [23], and further trials are needed to clarify this relationship. As shown in Table 2, the heterogeneity of devices, dosimetric parameters, and diagnostic criteria across trials is the principal reason why effect estimates differ between studies and why optimal protocols cannot yet be firmly established.

**Table 2:** Summary of the main systematic reviews, meta-analyses, and clinical studies of LLLT in TMD LLLT: low-level laser therapy; RCT: randomized controlled trial; VAS: visual analog scale; TENS: transcutaneous electrical nerve stimulation; NSAIDs: nonsteroidal anti-inflammatory drugs; GaAs: gallium arsenide; TMJ: temporomandibular joint; TMD: temporomandibular disorder

Study (year) [ref]	Design	Sample	Laser parameters	Comparator(s)	Main findings
Díaz et al. (2025) [16]	Systematic review of RCTs	44 RCTs; 1,816 patients	810-940 nm; 3-12 J/cm²	Occlusal splints, NSAIDs, TENS	~60-70% reduction in VAS pain and ~10-20% increase in mouth opening; benefits more sustained beyond four weeks; adverse effects rare and transient
Buduru et al. (2024) [18]	Systematic review (PRISMA)	7 studies (5 RCTs, 2 before-after); 324 patients	633-940 nm; 25-1,600 mW; 3-12 sessions, 1-3/week	Various	Consistent pain reduction and improved mandibular movement and muscle function; effective for muscle-related TMD, though protocols remain nonstandardized
Ren et al. (2022) [15]	Systematic review + network meta-analysis	27 RCTs; 969 patients	Red (632.8-672 nm) and infrared (780-904 nm; 910-1100 nm) bands compared	Placebo, TENS	910-1100 nm produced the greatest pain reduction vs placebo immediately after treatment (MD: 4.68; 95% CI: 3.08-6.28), not significant at one month; only 780-904 nm remained significant at one month; no significant difference between TENS and infrared laser bands
Carvalho et al. (2010) [10]	Clinical study (wavelength effect)	Patients with TMJ pain	Infrared 780-904 nm	-	Infrared wavelengths were more suitable owing to deeper tissue penetration; efficacy enhanced by higher energy-density protocols and a greater number of sessions
Sayed et al. (2014) [19]	Clinical study	20 TMD patients	GaAs 904 nm; 4 J/cm²; 6 sessions over 2 weeks	-	Significant reduction in tender points and joint sounds and improved jaw range of motion, maintained at the 6-month follow-up
Desai et al. (2022) [21]	Double-blind, placebo-controlled RCT	Patients with TMJ pain	LLLT vs sham	Placebo	Significant improvements in pain, maximum mouth opening, mandibular deviation, and articular clicking compared with placebo
Farshidfar et al. (2023) [20]	Updated systematic review of RCTs	40 studies; 1,927 patients	Photobiomodulation (varied)	Placebo	7 of 15 studies reporting maximum mouth opening showed a significantly greater improvement with photobiomodulation than with placebo
Mahammadli et al. (2026) [23]	RCT; two DC/TMD diagnostic subgroups (myofascial pain vs. disk displacement with reduction), each subdivided by energy density	100 patients; DC/TMD diagnosis, MRI-confirmed disk displacement	940 nm; 1.5 W (90 J) or 3 W (180 J); 9 sessions over 3 weeks	Soft diet-only control	Significant pain reduction at 1 and 6 months in all subgroups (myogenic and arthrogenic); comparable improvements in the soft-diet control group, with no clear superiority of LLLT over the control condition
Trindade et al. (2026) [22]	Randomized, controlled, blinded (placebo) trial	29 patients; DC/TMD Axis I and II	LED; 850 nm (infrared) + 660 nm (red); 6 sessions over 2 weeks	Placebo (inactive device)	Significant VAS pain reduction in the active LED group (p = 0.0009); no significant change in the placebo group; no difference in mouth opening between groups

Comparison with conventional therapies

LLLT has frequently been compared with the conservative treatments most commonly used for TMD, namely, occlusal splints, NSAIDs, and TENS. The systematic review of Díaz et al. reported that LLLT achieved pain relief comparable or superior to that obtained with these conventional options, while offering a more favorable tolerability profile [16]. In the network meta-analysis of Ren et al., both infrared LLLT and TENS reduced pain relative to placebo immediately after treatment, without a statistically significant difference between them; at one-month follow-up, only the 780-904 nm laser band remained significantly superior to placebo, whereas TENS did not [15]. These comparisons should nonetheless be interpreted with caution, as they rest on a limited number of active-comparator trials, generally of short duration and heterogeneous design, and few studies have evaluated the combination of LLLT with splint therapy or physiotherapy, which may act synergistically [4,18]. Rather than replacing established conservative measures, LLLT therefore appears best positioned as an adjunctive modality within a multimodal treatment plan, particularly when a rapid, non-pharmacological analgesic effect is desirable.

Safety, contraindications, and adverse effects

LLLT is widely recognized as a safe and well-tolerated noninvasive treatment modality for TMD [9,16]. A systematic review of 44 randomized clinical trials encompassing 1,816 participants reported no severe adverse events [16]. The most commonly observed side effects were transient erythema, occurring in 3% to 5% of cases, and mild local discomfort, reported in fewer than 2% of patients [16]. These findings confirm the favorable safety profile of LLLT, supporting its suitability for repeated use in patients with chronic TMD [16].

The nonablative and nonthermal mechanism of action, which is photochemical rather than thermal, accounts for the low incidence of tissue damage or undesirable effects [8]. Because low-level lasers do not evaporate or macroscopically alter tissue structure and do not produce a noticeable increase in tissue temperature, treatment is generally well accepted and comfortable for patients [8].

Several contraindications and precautions should nonetheless be observed. Because near-infrared laser beams are invisible yet can damage the retina, wavelength-appropriate protective eyewear should be worn by both the patient and the operator, in accordance with the safety class of the device and applicable laser-safety standards [24]. LLLT is contraindicated over the gravid uterus in pregnant women [8]. Photosensitive patients with epilepsy should be informed that low-frequency pulsed visible light may trigger a seizure [8]. Patients with pacemakers should not receive LLLT without careful consideration, and the therapy should be avoided in patients with chest pain [8]. Furthermore, although there are no absolute contraindications to LLLT, its application over or near malignant or premalignant lesions remains a matter of caution, given the theoretical concern that laser irradiation may promote cancer cell proliferation, an issue that current evidence has not fully resolved [25].

Most studies included in the literature utilized placebo or sham control groups and reported no significant adverse events related to laser application [8,16]. The therapeutic effects of LLLT were consistently associated with positive outcomes in pain reduction and functional improvement, without an accompanying increase in side effects compared to placebo [16]. In conclusion, LLLT offers a safe, noninvasive, and well-tolerated therapeutic option for TMD, with a favorable risk-benefit profile [8,9,16]. However, the lack of standardized protocols and the need for further long-term safety assessments remain important areas requiring investigation [16].

Limitations

This review has several limitations. First, it is narrative in design and does not include a formal risk-of-bias assessment or meta-analysis. Second, the available evidence is heterogeneous in terms of laser wavelength, power, fluence, number of sessions, application sites, and TMD diagnostic criteria. Third, many studies include small sample sizes and short follow-up periods. Therefore, the reported parameters summarized in Table 1 should be interpreted as clinically oriented guidance rather than definitive standardized recommendations.

## Conclusions

This narrative review suggests that LLLT may represent a safe adjunctive treatment for temporomandibular disorders, with reported benefits on pain, mouth opening, muscular tenderness, and joint sounds. However, the magnitude and consistency of these effects remain highly variable across studies, and this variability, rather than a predictable therapeutic response, is a central finding of the present review. The most frequently reported parameters include near-infrared wavelengths (810-1100 nm, most often 810-940 nm), energy densities of 3-12 J/cm², and 3-12 sessions over two to four weeks, with protocols exceeding four weeks associated with more sustained benefits; in the limited number of short-term active-comparator trials, outcomes were comparable or superior to those of occlusal splints, NSAIDs, and TENS.

However, heterogeneity in diagnostic criteria, dosimetric reporting, and study design limits the generalizability of these findings, and optimal parameters for specific TMD subtypes remain undefined. Future large-scale RCTs with standardized DC/TMD diagnoses and longer follow-up are needed. In the meantime, LLLT can be a valuable component of multidisciplinary conservative care when applied with careful attention to technical parameters and awareness of current methodological limitations.
